# The use of end-quintile comparisons to identify under-servicing of the poor and over-servicing of the rich: A longitudinal study describing the effect of socioeconomic status on healthcare

**DOI:** 10.1186/1472-6963-5-61

**Published:** 2005-09-09

**Authors:** Kate J Brameld, C D'Arcy J Holman

**Affiliations:** 1 Centre for Health Services Research, School of Population Health, The University of Western Australia, 35 Stirling Highway, Crawley, Western Australia, 6009

## Abstract

**Background:**

To demonstrate the use of end-quintile comparisons in assessing the effect of socio-economic status on hospital utilisation and outcomes in Western Australia.

**Methods:**

Hospital morbidity records were extracted from the WA Data Linkage System for the period 1994–99, with follow-up to the end of 2000. Multivariate modelling was used to estimate the effect of socio-economic status on hospital admission rates, average and total length of stay (LOS), cumulative incidence of readmission at 30 days and one year, and case fatality at one year.

**Results:**

The study demonstrated higher rate ratios of hospital admission in the more disadvantaged quintiles: rate ratios were 1.31 (95% CI 1.25–1.37) and 1.32 (1.26–1.38) in the first quintile (most disadvantaged) and the second quintile respectively, compared with the fifth quintile (most advantaged). There was a longer total LOS in the most disadvantaged quintile compared with quintile 5 (LOS ratio 1.24; 1.23–1.26). The risk of readmission at 30 days and one year and the risk of death at one year were also greater in those with greater disadvantage: the hazard ratios for quintiles 1:quintile 5 were 1.07 (1.05–1.09), 1.17 (1.16–1.18) and 1.10 (1.07–1.13) respectively. In contradiction to the trends towards higher hospital utilisation and poorer outcomes with increasing social disadvantage, in some MDC's the rate ratio of quintile 1:quintile 2 was less than 1, and quintile 4:quintile 5 was greater than 1. For all surgical admissions the most disadvantaged had a significantly lower admission rate than the second quintile.

**Conclusion:**

This study has shown that the disadvantaged within Western Australia are more intensive users of hospital services but their outcomes following hospitalisation are worse, consistent with their health status. Instances of overuse in the least disadvantaged and under use in the most disadvantaged have also been identified.

## Background

A recent review of the Australian literature unequivocally showed that the "disadvantaged" had higher mortality rates from most major causes of death, experienced more ill-health and were less likely to act to prevent disease or detect it at an asymptomatic stage [1,2]. Available evidence also suggests that the disadvantaged have experienced more hospital admissions and a higher number of medical consultations [3,4].

This study utilises the unique facility of the Western Australian Linked Data System to provide an overview of the effects of socio-economic status on hospital utilisation and outcomes throughout the State of Western Australia for each major diagnostic category, while adjusting for the effects of age, sex, Aboriginality, comorbidity, geography and possession of health insurance. In addition to providing this general overview of inequalities in hospital utilisation and outcomes in WA, the study also attempts to identify possible examples of under- and over-servicing. Examples of over/under servicing may be indicated, particularly where the procedure is discretionary and where other factors such as disease prevalence, demographic characteristics, medical resources and differing physician practice patterns have been accounted for. [5]

A particular asset of the study is the assignment of socioeconomic status at the level of the Census Collectors' District (CD), which consists of approximately 250 households in urban areas and fewer dwellings in a rural area. This is thus a far more accurate measure than has previously been possible at the postcode level. The data linkage system also enables us to present a greater range of hospital utilisation and outcomes measures and these are given for the complete range of diagnostic categories. In addition we have also been able to adjust for age, sex, aboriginality, accessibility, comorbidity and possession of private health insurance at the individual level.

The study allows areas of inequity to be identified, thus facilitating the development of policies and targeting of resources to address these inequalities.

## Method

All linked hospital morbidity and death records were selected from the WA Data Linkage System where a patient had any diagnosis in a specified major diagnostic category (MDC) during the period 1994–1999. For the study of hospital admissions and length of stay (LOS) only records with a diagnosis in the specified MDC between 1994 and 1999 were selected. For the study of cumulative risk of readmission within 30 days and 1 year and case fatality within one year, the patient's first record with a diagnosis of the specified MDC in the period 1994–1999 was selected (the index admission) along with the next admission record with any diagnosis that occurred within a 30 day or 1 year timeframe, as well as any linked death record.

The effect of socio-economic status on hospital admission rates was modelled using Poisson regression. All models were corrected for overdispersion of the data. Multiple linear regression was used to model average LOS of all admissions during the 12 months following index admission and cumulative LOS in the 12 months following index admission. LOS analyses were based on logarithm-transformed data because of skewness in the distribution. Age was squared to improve the model fit. The effects of the study variables on the cumulative risk of readmission within 30 days and 1 year and case fatality within one year were measured using Cox regression. Here the confounding effect of age was modelled using fractional polynomial regression[6]. In all cases, risk adjustment was made for age, sex, Aboriginality, comorbidity, social disadvantage and possession of health insurance. Comorbidity was measured using the Charlson Index, [7,8] which was adapted for use with ICD-10-AM. The allocation of medical and surgical episodes was performed on the basis of DRG. Patient transfers between hospitals were identified and were concatenated into one hospital episode, beginning with the first admission date and ending with the final separation date in the transfer set.

Socio-economic status was measured using the Australian Bureau of Statistics socio-economic indices for areas (SEIFA), specifically the index of relative disadvantage at the level of the census Collector's District (CD). The index of relative socioeconomic disadvantage was one of five SEIFA indices [9]. The indices were created by combining a range of variables using principal component analysis. For the index of relative disadvantage these included qualifications, income, unemployment, type of job, home ownership, one parent families, marital status, car ownership, school leaving age, Aboriginality, number of families per household and fluency in English [9]. In this study SEIFA values were divided into quintiles with 20% of the population in each quintile.

Locational disadvantage was measured using the accessibility/remoteness index of Australia (ARIA),[10]. The ARIA index calculates remoteness based on the distance by road to specified service centres. There are four categories of service centres depending on size, the smallest having a population of 5,000. Every populated locality gets a score between 0 and 12 and these are aggregated into five categories ranging from highly accessible to very remote. For the purpose of this study, the five categories were further collapsed into three; remote, accessible and highly accessible, due to the small number of patients in the less accessible areas.

The measures of locational and social disadvantage were matched to the hospital morbidity records using the CD of residence of the patient as a linkage key. CD's were derived from a geographical point defined by a specific longitude and latitude (a geocode). All patient addresses in the hospital morbidity database were geocoded commencing from records collected in 1993, prior to which the records contained insufficient address information. Even so, approximately 20% of records from 1993 onwards were unable to be geocoded due to incomplete address information. In some sparsely populated areas, CDs for records with missing geocodes could be allocated from the postcodes. In other cases it was possible to allocate a CD from the patient's other linked records, provided that the patient had maintained the same residential postcode and a period of less than five years had elapsed between the admissions. In most of the remaining 16% of records, where a CD was still missing at this stage, the SEIFA and ARIA codes were allocated on the basis of postcode. This left a small proportion of records (1.5%) still without SEIFA and ARIA codes, because not all postcodes were assigned a SEIFA code during the 1996 census. These records were excluded from the analysis.

Possession of private health insurance was allocated on the basis of the payment classification variable having the value of 'private insured'. A patient only required one record with this payment classification to be recorded as privately insured for all analyses in the study. This is based on the assumption that some patients did not always declare or utilise their private health cover, depending on the circumstances of the admission, such as whether emergency or elective treatment was needed; anticipated levels of copayment as a private patient; and whether a privately insured classification was really necessary to secure the patient's choice of doctor. This enabled us to gain an overview of how insurance status affected the patient's entire treatment history, as distinct from the times when they elected to use their private insurance.

Population denominators for the Poisson regression were based on data from the ABS CDATA96 [11]. The proportion of the population with health insurance was taken from the ABS Health Insurance Survey of Australia [12]. The distribution of patients in the population in each comorbidity category was taken as the average distribution across all the MDCs. That is, 72% of patients had no comorbid condition, 23% had one or two comorbidities, 3% had three of four comorbidities and 2% had more than four comorbidities.

The study involved a total of 1,061101 patients admitted over the five year period. Descriptive information about these patients is given in table 1.

**Table 1:** Descriptive statistics for Patients in the Study

**Variable**		**Number**	**Percentage**
Agegroup	0–4	188827	17.7
	5–9	41760	3.9
	10–14	36173	3.4
	15–19	57212	5.4
	20–24	69732	6.6
	25–29	81310	7.7
	30–34	82977	7.8
	35–39	73647	6.9
	40–44	64366	6.1
	45–49	62323	5.9
	50–54	55034	5.2
	55–59	46438	4.4
	60–64	42300	4.0
	65–69	42244	4.0
	70–74	40292	3.8
	75+	76987	7.3
			
Sex	Male	484935	45.7
	Female	576166	54.3
			
No. of comorbidities	0	763993	72.0
	1–2	244053	23.0
	3–4	31833	3.0
	5+	21222	2.0
			
SEIFA category	1 – Extreme disadvantage	211371	19.9
	2 – disadvantage	243432	22.9
	3 – average	225003	21.2
	4 – advantaged	184621	17.4
	5 – extreme advantage	196674	18.5
			
ARIA category	1 – Remote	80318	7.6
	2 – Accessible	139986	13.2
	3 – Highly accessible	840797	79.2
			
Aborginality	Yes	40748	3.8
	No	1020353	96.2
			
Possession of health insurance	Yes	348396	32.8
	No	712705	67.2
			
Total persons		1061101	

## Results

The overall admission rates showed a clear difference between the most and least disadvantaged groups, but there was little difference between the two most disadvantaged quintiles and between the two least advantaged quintiles (table 2). A similar picture was seen for medical admissions, whereas for surgical admissions the most disadvantaged (first quintile) had a significantly lower admission rate than the second quintile.

**Table 2:** The effect of socio-economic status on hospital utilisation and outcomes

	Most disadvantaged								Least disadvantaged
	
	Ratio q1:q5	95% CI	Ratio q2:q5	95% CI	Ratio q3:q5	95% CI	Ratio q4:q5	95% CI	Baseline – q5
Admission rate – all	1.31	(1.25–1.37)	1.32	(1.26–1.38)	1.20	(1.15–1.25)	0.98	(0.94–1.03)	1.00
Admission rate – medical	1.39	(1.33–1.45)	1.35	(1.29–1.41)	1.20	(1.15–1.26)	0.97	(0.92–1.01)	1.00
Admission rate – surgical	1.12	(1.08–1.15)	1.21	(1.18–1.26)	1.13	(1.09–1.16)	0.98	(0.94–1.01)	1.00
Average LOS	1.04	(1.04–1.05)	1.03	1.03–1.04	1.03	(1.03–1.04)	1.03	(1.02–1.03)	1.00
Total LOS	1.24	(1.23–1.26)	1.14	(1.13–1.16)	1.12	(.10–1.13)	1.08	(1.07–1.10)	1.00
Readmission at 30 days	1.07	(1.05–1.09)	1.05	(1.03–1.06)	1.03	(1.01–1.05)	1.01	(0.99–1.03)	1.00
Readmission at 1 year	1.17	(1.16–1.18)	1.11	(1.10–1.12)	1.07	(1.06–1.08)	1.05	(1.04–1.05)	1.00
Case fatality at 1 year	1.10	(1.07–1.13)	1.05	(1.03–1.08)	1.03	(1.01–1.06)	1.04	(1.01–1.07)	1.00

Socioeconomic status had little effect on average length of stay, but total length of stay decreased with increasing advantage. The risk of readmission within 30 days and 1 year and case fatality within 1 year decreased with increasing social advantage (table 2).

Examination of medical admission rate ratios at the MDC level (table 3) showed that in most cases (9 out of 17 MDCs), those in the most disadvantaged quintile had the highest risk of admission or their risk was similar to those in the second quintile (4 out of 17 MDCs). There were a further four MDCs where the risk in the most disadvantaged was lower than for quintile 2. With regards to the most advantaged quintile, their risk of admission was higher than that for quintile 4 for four out of 17 MDCs, similar to quintile 4 in eight out of 17 MDCs and lower than quintile 4 for five out of 18 MDCs.

**Table 3:** The effect of socio-economic status on hospital admission rates for medical DRGs

MDC		Quintile 1:quintile 2		Quintile 5 :quintile 4	
		
		Rate ratio	95% CI	Rate ratio	95% CI
1	Nervous	1.05	(1.00 – 1.11)	0.96	(0.90–1.02)
2	Eye*	1.21	(1.15 – 1.28)	1.06	(0.99–1.15)
3	ENTM	**0.87**	(0.83 – 0.91)	**1.17**	(1.12–1.24)
4	Respiratory	1.15	(1.10 – 1.20)	0.92	(0.86–0.99)
5	Circulatory	1.02	(0.99 – 1.06)	0.96	(0.92–1.00)
6	Digestive	0.93	(0.89 – 0.96)	**1.11**	(1.06–1.15)
7	Pancreas*†	1.23	(1.16 – 1.29)	0.90	(0.83–0.96)
8	Musculoskeletal	1.01	(0.97 – 1.05)	1.01	(0.97–1.06)
9	Skin	1.19	(1.14 – 1.26)	1.06	(0.99–1.14)
10	Endocrine*†	1.23	(1.14 – 1.33)	0.93	(0.83–1.05)
11	Kidney*†	1.47	(1.32 – 1.64)	0.65	(0.57–0.75)
12	Male repro†	0.92	(0.85 – 1.00)	**1.12**	(1.04–1.21)
13	Female repro†	1.18	(1.09 – 1.29)	1.06	(0.96–1.16)
16	Blood	0.93	(0.89 – 0.98)	0.90	(0.85–0.95)
17	Neoplastic	**0.89**	(0.85 – 0.92)	**1.11**	(1.07–1.16)
21	Injuries	1.13	(1.08 – 1.18)	1.02	(0.96–1.08)
22	Burns*	1.18	(1.09 – 1.28)	0.90	(0.83–0.98)
	Total	1.03	(0.99 – 1.07)	1.03	(0.99–1.09)

Bold text denotes over or underservicing

* questionable convergence of poisson model

† restricted subgroups to allow model convergence

MDC 7 age ≥ 20 years

MDC 10 age ≥ 25 years and 0–2 comorbid conditions

MDC11 age ≥ 30

MDC12 age range 30–64 years and sex male

MDC13 age range 30–64 years and sex female

Analysis of surgical admission rate ratios (table 4) highlighted seven out of 17 MDCs where quintile 1 had a lower risk of admission than quintile 2, six MDCs where the risk was similar and four MDCs where the highest risk was seen in quintile 1. The most advantaged had a higher risk of admission in four out of 17 MDCs, a similar risk in 11 out of 17 MDCs and s lower risk in 2 out of 17 MDCs.

**Table 4:** The effect of socio-economic status on hospital admission rates for surgical DRGs

MDC		Quintile 1:quintile 2		Quintile 5: quintile 4	
		
		Rate ratio	95% CI	Rate ratio	95% CI
1	Nervous	0.99	(0.95 – 1.03)	0.92	(0.88–0.96)
2	Eye	**0.90**	(0.88 – 0.93)	1.09	(1.06–1.13)
3	ENTM	0.91	(0.88 – 0.94)	1.02	(0.98–1.05)
4	Respiratory*	1.09	(1.04 – 1.14)	1.03	(0.97–1.09)
5	Circulatory	0.95	(0.91 – 0.98)	0.96	(0.93–1.00)
6	Digestive	**0.90**	(0.87 – 0.94)	1.01	(0.98–1.05)
7	Pancreas†	1.01	(0.97 – 1.05)	0.97	(0.92–1.02)
8	Musculoskeletal	**0.89**	(0.86 – 0.92)	1.04	(1.01–1.08)
9	Skin	**0.88**	(0.84 – 0.92)	1.08	(1.03–1.13)
10	Endocrine†	0.94	(0.88 – 1.01)	1.07	(1.00–1.14)
11	Kidney*†	1.14	(1.06 – 1.23)	0.86	(0.78–0.94)
12	Male repro†	0.95	(0.89 – 1.02)	1.06	(0.98–1.13)
13	Female repro†	0.97	(0.92 – 1.03)	1.00	(0.94–1.05)
16	Blood*	0.97	(0.91 – 1.03)	0.95	(0.89–1.02)
17	Neoplastic*	**0.90**	(0.85 – 0.96)	**1.17**	(1.10–1.25)
21	Injuries*	1.07	(1.02 – 1.11)	0.97	(0.92–1.03)
22	Burns*	1.16	(1.07 – 1.26)	0.85	(0.78–0.92)
	Total	0.92	(0.89 – 0.95)	1.02	(0.99–1.06)

Bold text denotes over or underservicing

* questionable convergence of poisson model

† **restricted subgroups to allow model convergence**

End-quintile comparisons of medical and surgical admission rates was used to highlight possible instances of under- and overservicing, whereby the admission rate in the first quintile was at least 10% less than that in the second quintile (ie, rate ratio ≤ 0.90) or the admission rate in the fifth quintile was more than 10% greater than in the fourth quintile (ie. rate ratio ≥ 1.10). Examples of where this occurred are highlighted in tables 3 and 4 for medical and surgical admissions respectively. The most common procedures (those responsible for ≥ 5% of admissions) within the MDCs where the possibility of under- or overservicing was identified are shown in tables 5 and 6.

**Table 5:** Most common procedures in the MDCs for which underservicing of the socially disadvantaged occurred

**MDC**	**ICD-9-CM code**	**Description**
2	13.41	Phacoemulsification and aspiration of cataract
	13.59	Other extracapsular extraction of lens
3	23.19	Other surgical extraction of teeth
	23.13	Surgical extraction of two or more teeth
	23.09	Forceps extraction of other tooth
6	47.0	Appendectomy
	49.46	Excision of haemorrhoids
	53.00	Unilateral repair of inguinal hernia
8	80.6	Excision of semilunar cartilage of knee
	81.47	Other repair of knee
9	86.3	Other local excision or destruction of lesion or tissue of skin and subcutaneous tissue
	85.21	Local excision of lesion of breast
17	99.25	Chemotherapy
	41.31	Biopsy of bone marrow
	40.11	Biopsy of lymphatic structures
	99.04	Transfusion of packed cells

**Table 6:** Most common procedures in the MDCs for which overservicing of the socially advantaged occurred

**MDC**	**ICD-9-CM code**	**Description**
3	23.19	Surgical extraction of teeth
	23.13	Surgical extraction of two or more teeth
	23.09	Forceps extraction of tooth
6	45.16	Oesophagogastroduodenoscopy with closed biopsy
	45.23	colonoscopy
	45.42	Colonoscopic polypectomy
	45.13	Other endoscopy of small intestine
	45.25	Closed biopsy of large intestine
12	63.73	Vasectomy
	57.32	Other cystoscopy
	60.11	Closed biopsy of prostate
17	99.25	Chemotherapy
	41.31	Biopsy of bone marrow
	40.11	Biopsy of lymphatic structures

## Discussion

Medicare, Australia's universal health insurance scheme was designed to ensure that all Australians had equal access to health care [13]. Equity of access to healthcare in Australia is generally accepted to mean "equal access to equal care for equal need", while recognising that the underprivileged may require more access to more care for the same health problem [14,15]. Numerous studies have shown that those of lower socio-economic status have higher mortality and morbidity rates and their behaviour is more likely to be detrimental to their health; for example, they are more likely to have a poorer diet, be less physically active, drink alcohol to excess and smoke more cigarettes [2]. As a result we would expect them to have a greater requirement for health services.

Our study demonstrated higher hospital admission rates in the disadvantaged. A number of studies have shown that those of low socio-economic status, as measured by various indicators, most commonly income, have a greater risk of hospitalisation than those of high socio-economic status [16-18]. In addition it has been shown that the socio-economic gradient is much greater for medical than surgical admissions as we have also shown (table 1), [16]. However, our study found some anomalies to the general pattern whereby the admission rate was higher in quintile 2 than in quintile 1 (most disadvantaged) and/or the admission rate in the quintile 5 was higher than in quintile 4. This was particularly noticeable for surgical admissions, where the admission rate in quintile 2 was significantly higher than that in quintile 1 (Table 1). This phenomenon is indicative of underservicing in the most disadvantaged and overservicing in the least disadvantaged groups in relation to the segment of the population of nearest socioeconomic status. Further research is required to identify the extent to which this may represent differing rates of access to care or different methods of patient management. It is unlikely to be a result of underlying disease prevalence, which in many cases is higher in the socially disadvantaged. [2]

In some cases, for example chemotherapy, apparent over- or underservicing may have occurred as a result of some patients being treated as day patients (and thus were not included in our hospital morbidity data). Nevertheless, this would still represent a differential pattern of treatment according to socioeconomic group. The cost of surgical extraction of teeth is not covered by Medicare and this is clearly resulting in differential treatment according to socioeconomic status. In other cases, it is likely that those in higher socio-economic groups are better able to negotiate their way through the health system to achieve their desired outcome [19]. While this study has adjusted for possession of private health insurance, the prevalence of which increases with increasing social advantage, more advantaged groups are also better able to pay for procedures in the private sector, as and when necessary [20].

The study showed that hospital admission rates in the most disadvantaged were up to 35% higher than in the most advantaged. In contrast, age-standardised mortality rates for those aged under 65 in Australia during 1998–2000 were 50–90% higher for the most disadvantaged, dependant on age-group and sex [21]. Premature mortality rates have been suggested as the best single indicator of health status that reflects the need for health care and this suggests that the increased admission rate in the most disadvantaged does not match their increased need [22].

The study also demonstrated that the most disadvantaged had a significantly longer total LOS. Canadian data examining all hospitalisations in residents of Winnipeg from 1989–1996, have shown a strong association between length of stay for admissions of 59 days or less and socio-economic status, poorer patients staying in hospital for up to 2.4 times longer than the most affluent patients [18]. A similar result was reported by Epstein et al in a study of nearly 17,000 patients admitted to Massachusetts hospitals during 1987 [23]. They found that patients of lowest socio-economic status had stays of 3–30% longer than those of higher status in 14 out of 15 comparisons when adjusting for age, severity of illness and DRG.

Socio-economic status was also shown to have a significant effect on the outcome measures, risk being greatest in those with greatest disadvantage. However, the effect on the risk of readmission was greater at 1 year than 30 days. This is important because early readmission rates are indicative of possible deficiencies in the process of inpatient care, whereas readmission rates in general may be more an effect of disease progression or the onset of new disease [24]. There were few studies examining the effect of socio-economic status on readmission rates. Weissman et al conducted a study of nearly 12,000 patients adjusted for age, gender, hospital, severity of illness and DRG and found that those of low SES had a greater risk of readmission within 60 days [25].

The effect of socio-economic status on case fatality has mainly been studied following myocardial infarction. Macintyre *et al *found that the more deprived were more likely to die within 30 days of their myocardial infarction. Alter et al found a similar relationship with one year mortality the Salomaa *et al *also found that case fatality at 28 days and one year was highest in those with low income and education, consistent with our results [26-28].

Increased use of hospital services by the more disadvantaged in our population reflect their greater health need and, in many cases, also a greater severity of illness [23,29]. Weissman et al suggested that greater difficulty in accessing ambulatory care post-discharge, inability to afford recommended therapies and possible non-compliance or misunderstanding of physicians orders may increase the risk of readmissions [25].

## Conclusion

This study has shown that the socially disadvantaged within Western Australia are more intensive users of health services and their outcomes following hospitalisation are worse. Comparison of hospital admission rates and premature mortality rates suggest that the increased admission rates in the most disadvantaged are not sufficient to account for their increased need. Some examples of overuse in the least disadvantaged and under use in the most disadvantaged were also identified. These factors together with the discrepancy in the gradient between medical and surgical admissions are suggestive of inequity in treatment between socio-economic groups, specifically the greatest and the least disadvantaged.

The WA data linkage system provides an effective mechanism for ongoing monitoring of equity in hospital utilisation and outcomes. Further research is required to identify the need for health services according to socio-economic groups and to identify how services can be made more easily accessible for these groups.

## Competing interests

The author(s) declare that they have no competing interests.

## Authors' contributions

KJB participated in the conception and design of the study, performed the analysis and drafted the paper. CDJH participated in the conception and design of the study and in revising the paper.

## Pre-publication history

The pre-publication history for this paper can be accessed here:


